# Recent Progress in Treating Airway Mucus Hypersecretion by Targeting the Epidermal Growth Factor Receptor Signaling Pathway

**DOI:** 10.1002/pdi3.70007

**Published:** 2025-05-10

**Authors:** Yuanyuan Zhang, Fuping Yang, Chunyu Yan, Yu Deng, Enmei Liu

**Affiliations:** ^1^ Department of Respiratory Medicine Ministry of Education Key Laboratory of Child Development and Disorders Chongqing Key Laboratory of Pediatrics Children's Hospital of Chongqing Medical University National Clinical Research Center for Child Health and Disorders Chongqing China

**Keywords:** airway mucus hypersecretion, drug therapy, epidermal growth factor receptor (EGFR)

## Abstract

Asthma, chronic pulmonary obstructive disease (COPD), cystic fibrosis, and acute respiratory infections are severe respiratory conditions that significantly contribute to global morbidity and mortality. Airway mucus hypersecretion is an important common pathophysiological and clinical manifestation of these diseases and is closely associated with adverse clinical outcomes. At present, the management of airway mucus hypersecretion lacks a viable clinical intervention. However, the revelation that the EGFR signaling pathway acts as a convergent intracellular pathway for many inflammatory mediators indicates that it exhibits the greatest potential for inhibiting excessive mucus production. This review provides a comprehensive overview of the role of EGFR in airway mucus hypersecretion and the therapeutic progress of targeting it, intending to improve clinicians' understanding of the mechanism and offer insights and recommendations for developing novel pharmaceutical interventions to regulate mucus secretion.

## 1 Introduction

The mucus that coats the inner lining of the airway is a crucial component of the innate immune response of the lungs, serving to safeguard the respiratory system from potential injury caused by inhaled pathogens, particles, and toxic chemicals [1]. Normally, a moderate quantity of mucus secretion protects the airways. However, many patients with respiratory diseases, including chronic obstructive pulmonary disease (COPD), asthma, cystic fibrosis (CF), and infection, have mucus hypersecretion, which is a common pathophysiological feature of these diseases, clinically manifested as cough and sputum [2]. Mucus hypersecretion not only triggers a range of symptoms but also accelerates disease progression and significantly impacts clinical outcomes. As research has advanced, its significance has been gradually recognized by official agencies [3, 4].

COPD stands as the third leading cause of death worldwide, with 3.23 million fatalities reported in 2019 [5]. Approximately 50% of patients with COPD exhibit airway mucus hypersecretion, which has been proven linked to impaired lung function and a decline in quality of life [6]. Moreover, patients with COPD with this condition face a mortality risk, that is, 3.5 times higher compared to those without mucus hypersecretion [7]. In patients with asthma, airway obstruction caused by excessive mucus secretion can lead to sudden death [8]. Additionally, widespread mucus plugging is consistently detected at autopsy in patients with fatal asthma [9]. Thus, understanding and managing mucus hypersecretion is crucial for improving outcomes.

Currently, there is no specific treatment for airway mucus hypersecretion. Conventional therapies, including bronchodilators and mucolytics, primarily offer symptomatic relief, but their effectiveness in severe cases is often limited. Additionally, these medications are associated with significant side effects, such as heart rate abnormalities, visual disturbances, urinary retention, and metabolic disorders. Given these limitations, there is a pressing need to identify new therapeutic targets and develop more effective strategies to manage mucus hypersecretion.

Recent research has revealed that [4] the epidermal growth factor receptor (EGFR) is an essential component of the signal transduction pathway of airway mucus hypersecretion and it offers the greatest potential for inhibition of excessive mucus production [10]. In this article, we will review the role of the EGFR pathway in airway mucus hypersecretion and therapeutic advances using it as a drug target.

## 2 Mucus and Mucin

In the normal human airway, the surface of the airway epithelial cells is covered with a thin layer of liquid called airway surface liquid (ASL) that can prevent water loss and remove inhaled dust, microorganisms, and other harmful substances [11]. ASL is divided into two layers based on its properties and functions, namely the periciliary liquid layer (PCL) and the mucus layer (ML). The combination of these two sublayers forms a “gel‐on‐brush” structure [12].

Water (95%), proteins, ions, and macromolecules are the primary constituents of mucus. Mucins, which are highly O‐glycosylated and contain high levels of serine and/or threonine, are the primary macromolecular of mucus. The mucins can be classified into two categories: gel‐forming mucins and transmembrane mucins [13]. Gel‐forming mucins share a similar structure to transmembrane mucins and the difference is that they are secreted instead of being tethered to a membrane. Different mucins possess unique roles and exhibit site‐specific expression patterns. For instance, MUC5AC is exclusively expressed in goblet cells (GCs), whereas MUC5B is mostly expressed in mucous cells of the submucous glands [6], and these two proteins are the most important mucins.

Mucins are viscoelastic and adhesive, and their rheological properties allow mucus to trap pathogenic microorganisms, foreign particles, and other harmful substances contained in the inhaled air, then transport them along the respiratory tract to the outside of the lungs by ciliary beating and finally remove them by swallowing or coughing. The whole process is called mucociliary clearance (MCC) [11].

## 3 Airway Mucus Hypersecretion

Airway mucus hypersecretion is a complex pathophysiological process in which multiple inflammatory cells, inflammatory mediators, and signaling pathways are involved. Most patients diagnosed with COPD exhibit the pathophysiological hallmark of airway mucus hypersecretion [2]. Given the well‐recognized negative impact of airway mucus hypersecretion in these patients, the addition of expectorant therapy is critical in these patients.

In addition to traditional expectorant drugs (hypertonic saline aerosol, guaifenesin, and bronchodilators) [2], the reduction of mucin gene (*MUC*) expression based on signaling pathways to inhibit excessive mucus production has become an emerging therapeutic approach in recent years. There are many signaling pathways involved in airway mucus hypersecretion, including tumor necrosis factor‐α signaling, p38 mitogen‐activated protein kinase (MAPK) pathway, and EGFR‐mediated signaling pathways. With the deepening of relevant research, it is currently believed that the EGFR pathway is a convergent pathway implicated in the hyperplasia of GCs and the upregulation of *MUC5AC* gene expression triggered by oxidative stress and the Th2‐type cytokines [14]. Therefore, EGFR has attracted much attention as a promising potential target.

## 4 EGFR Structure and Activation

EGFR, also referred to as ErbB1 and HER1, which is the expression product of the proto‐oncogene *erbB1*, belongs to the type I transmembrane receptor tyrosine kinase (RTK) superfamily. Other family members include ErbB2 (Neu/HER2), ErbB3 (HER3), and ErbB4 (HER4). Studies have found that ErbB receptors play a crucial role in cell signaling, particularly in regulating cell growth and differentiation. Dysregulation of ErbB receptors, often through mutations or overexpression, such as EGFR, is closely linked to tumor development and progression [15]. Targeting the EGFR receptor through precision therapy, such as tyrosine kinase inhibitors (TKIs), has become one of the most important therapeutic modalities in cancer treatment.

**FIGURE 1:**
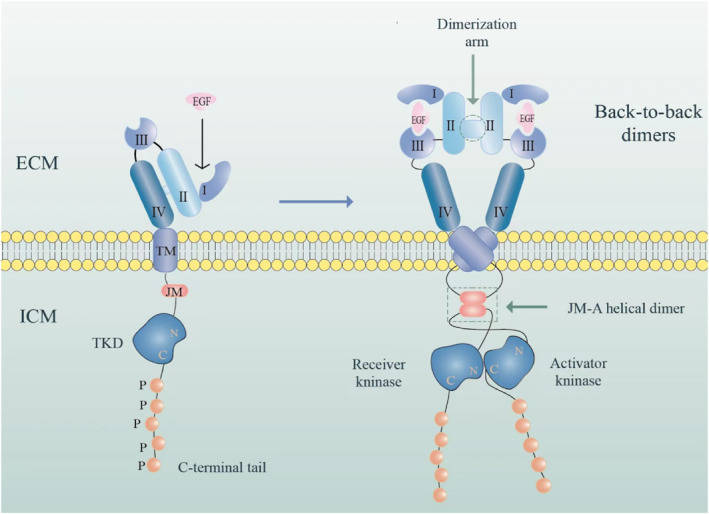
Cartoon of the EGF‐induced receptor dimerization process. Left: an EGFR monomer. Right: a ligand‐bound back‐to‐back dimer. Three structural components: the extracellular module (ECM), transmembrane (TM) domain, and intracellular module (ICM). The epidermal growth factor receptor (EGFR) consists of three main parts: ECM, TM, and ICM. The ECM, which binds ligands, has four subregions (I–IV). The ICM is a cytoplasmic carboxyl‐terminal region with a protein kinase domain, which contains three subregions, namely the tyrosine kinase domain (TKD), the juxtamembrane (JM) region, and a C‐terminal tail. EGF binding induces dimerization, resulting in a TM dimer, an antiparallel JM helical dimer, and a TKD dimer. This activates the kinase activity of the intracellular domain, leading to autophosphorylation and recruitment of downstream signaling molecules, thereby completing signal transduction.

EGFR is a transmembrane glycosylated protein with 1186 amino acids and three structural components: the extracellular module (ECM), the transmembrane (TM) domain, and intracellular module (ICM) [16]. The ECM contains the N‐terminus (ligand‐binding region) related to accepting external signals and is composed of four subregions I, II, III, and IV (or correspondingly called L1, S1/CR1, L2, and S2/CR2 subregions). The TM has a helical structure and is a hydrophobic region, anchored on the cell membrane. The ICM is a cytoplasmic carboxyl‐terminal region with a protein kinase domain, which contains three subregions, namely the tyrosine kinase domain (TKD), the juxtamembrane (JM) region, and a C‐terminal tail. EGF‐induced ligand‐receptor binding can form a back‐to‐back dimer, which are formed by the interconnection of a TM dimer, an antiparallel JM (JM‐A) helical dimer, and TKD dimer [17] (Figure 1).

The activation of EGFR can occur through two separate pathways: ligand‐dependent and ligand‐independent EGFR tyrosine phosphorylation [18]. In ligand‐dependent EGFR tyrosine phosphorylation, EGFR can be activated by binding to EGF family members such as EGF, transforming growth factor‐alpha (TGFA), and heparin‐binding EGF‐like growth factor (HBEGF) [19]. In epithelial cells, EGFR ligands are anchored on the cell membrane. Before they can bind to EGFR, they undergo a process of cleavage by some metalloproteinases (e.g., human neutrophil elastase or ADMA17) and they are eventually released in a mature form of about 50 amino acids to bind EGFR.

The activation process of EGFR after ligand binding can be divided into three steps: (1) Prior to EGFR binding to the ligand, both the extracellular region and the intracellular tyrosine kinase region of EGFR exist in a self‐repressed conformation [17]. Ligand binding to EGFR causes dimerization of the extracellular region of EGFR, bringing the intracellular region closer together and leading to the formation of an asymmetric dimer in the tyrosine kinase region. (2) This dimer activates the kinase region, resulting in tyrosine phosphorylation in both intracellular C‐terminal regions, by a mechanism known as “The Rotation Model” [20], in which the EGFR is twisted around the transmembrane region as an axis, causing the intracellular structural domain to conform to the kinase active region and phosphorylating the receptor tyrosine. (3) The phosphorylation of tyrosine residues of EGFR can be recognized by downstream proteins containing Src homology 2 (SH2) domains and phosphotyrosine‐binding (PTB) domains, which initiates a series of cascade reactions and transmits the information to the nucleus, causing the corresponding biological effects. EGFR activates multiple downstream signaling cascades, including the JAK/STAT signaling pathway involved in immune regulation, the Ras/Raf/MEK/ERK pathway (MAPK/ERK pathway) involved in cell proliferation and survival, and the PI3K/AKT/mTOR pathway involved in the regulation of cellular proliferation, differentiation, and survival. In addition, EGFR tyrosine phosphorylation can be activated by a ligand‐independent mechanism called “EGFR transactivation”, which is reported to be in response to oxidative stress that can be produced by cigarette smoke and activated neutrophils.

## 5 EGFR Activation Mediates Mucus Hypersecretion

Studies have found that compared with healthy people, the expression of EGFR and its ligands in airway epithelial cells of patients with CF, asthma, and COPD tends to be higher, and this alteration was positively correlated with mucus production and proliferation of airway epithelial GCs.

On one hand, EGFR activation is associated with airway goblet cell hyperplasia (GCH). Chronic airway inflammatory diseases, such as COPD, asthma, bronchiectasis, and CF, are characterized by a significant increase in the number of GCs in the airway epithelium, which serves as the structural basis for the airway mucus hypersecretion. Studies have shown that viral infection can also activate the EGFR signaling pathway, prompting the differentiation of ciliated cells into GCs, resulting in hypersecretion of airway mucus [21]. It is worth noting that the increased number of GCs is not due to GCH, but rather to the differentiation of EGFR‐containing granule‐free secretory cells in the airway epithelium into GCs precursors, which are then further converted into GCs.

**FIGURE 2:**
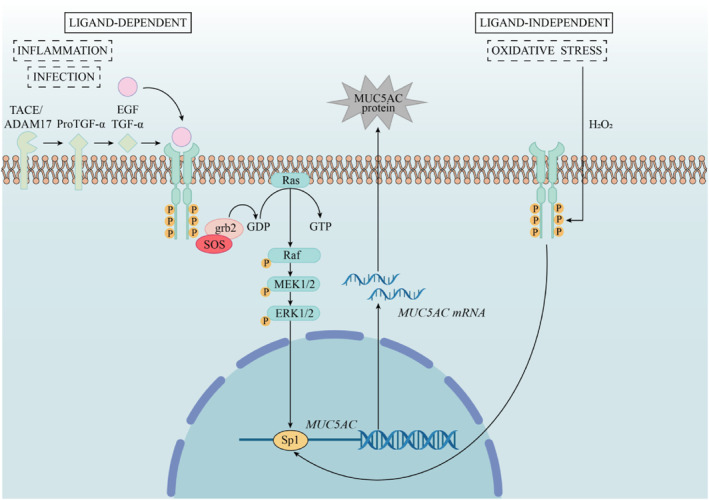
Schematic demonstrating the EGFR activation mediated mucus hypersecretion pathway. In the ligand‐dependent EGFR activation, EGF binding causes EGFR dimerization, which activates the Ras/Raf/MEK/ERK (MAPK) signaling pathway, transmitting signals to the nucleus and promoting *MUC* gene transcription, increasing mucin synthesis. Under oxidative stress, EGFR can be activated without a ligand. Sp1, a key transcription factor, enhances the expression of *MUC2* and *MUC5AC* genes and upregulates EGF and TGF‐α, amplifying mucin synthesis through positive feedback.

On the other hand, EGFR activation plays a key role in airway mucin synthesis. According to reports by Takeyama et al., EGFR immunoreactivity is positively correlated with MUC5AC‐positive staining area [22]. Oxidative stress, smoking, allergens, bacterial infection, mechanical injury, cytokines, and activated inflammatory cells can stimulate EGFR activation in two different pathways, ligand‐dependent or ligand‐independent, leading to increased mucin synthesis (Figure 2). The Ras/Raf/MEK/ERK (MAPK) signaling is the most prominent signaling pathway leading to the increased expression of airway MUC after EGFR activation.

In the ligand‐dependent EGFR activation, EGFR is phosphorylated on tyrosine residues upon binding to the corresponding ligand, which enables docking proteins such as Grb2 to bind to EGFR. At the same time, Grb2 binds to the guanine nucleotide exchange factor SOS and then SOS binds to Ras‐GDP, which can replace GDP with GTP to activate Ras protein. Activated Ras then activates the Raf kinase. After being activated, Raf phosphorylates and activates MEK. The activated MEK then phosphorylates and activates ERK. The MAPK pathway allows extracellular signals to be transmitted to the nucleus and activates *MUC* gene transcription, resulting in increased mucin synthesis in airway epithelial cells. Notably, studies have found that neutrophils play a key role in EGFR‐dependent mucus hypersecretion through multiple mechanisms [23].

The Sp1 is considered to be an important transcription factor involved in the induction of *MUC5AC* gene expression. The SP1 transcriptional regulatory sequence exists in the promoters of many viruses and cells. The SP1 transcription factor contains adjacent zinc‐finger motifs, has specific DNA binding activity, and is located in downstream of the EGFR pathway. Recently, some scholars reported that *MUC2* and *MUC5AC* are the target genes of EGFR ligands, and the up‐regulation of these two genes is accompanied by an increase in the opening of the EGFR/Ras/Raf extracellular signal‐regulated kinase pathway. Moreover, Sp‐1 plays a role in their activation. Sp‐1 not only promotes the expression of *MUC2* and *MUC5AC* but also upregulates the expression of EGF and TGF‐α, which greatly enhances this effect in the form of positive feedback [24].

Under normal conditions, activation of EGFR requires binding to its ligand to initiate intracellular signal transduction. However, under oxidative stress conditions, EGFR can be activated even in the absence of a ligand. This ligand‐independent activation may result from oxidative stress‐induced free radicals directly affecting EGFR, altering its conformation, thereby enabling it to trigger intracellular signaling pathways, and thereby inducing *MUC5AC* mRNA expression.

Moreover, studies have found [25] that the activation of EGFR in GCs of patients with COPD can downregulate the transcription factor FOXA2 and can also reduce the expression of another transcription factor involved in GC differentiation and ETS factor. The reduction of the expression of the above two transcription factors further promotes mucin production.

## 6 Advances in Drug Therapy Targeting EGFR

EGFR activation plays a central regulatory role in airway mucus hypersecretion, so its antagonists are a promising class of drugs for mucus hypersecretion therapy. In recent years, EGFR‐targeted drugs have been widely studied in tumor therapy, and there are two types of drugs that used to inhibit the EGFR family, namely monoclonal antibodies (MAbs) and small molecule TKIs.

MAbs can bind to the extracellular domain of EGFR and block its activation. In a recent study by Chen et al. [26], a single‐chain antibody fragment (scFv) against EGFR was genetically engineered and modified at the N‐terminal end of the human ferritin H‐chain (FTH1) to construct Anti EGFR scFv: FTH1/FTH1 nanoparticles. This nanoparticle can effectively alleviate asthma‐related symptoms in the asthmatic mouse model and inhibit GCH and mucus secretion in the lung tissue of asthmatic mice. Targeted EGFR nanotherapy offers a promising approach for managing hypersecretion, but there is a potential for its severe side effects due to nonspecificity, thus requiring further optimization of nanoparticle delivery systems and comprehensive evaluation in clinical trials.

TKIs, which include gefitinib, erlotinib, and afatinib, are a class of small‐molecule drugs that enter cells to competitively bind to the ATP site on the tyrosine residues of the receptor and prevent the activation of it. Takeyama et al. reported [27] that pretreatments with the EGFR TKI BIBX1522 in an asthma model can prevent the production of GCs. In the study of Hewson et al. [28] by injecting an EGFR inhibitor (AG1478) into RV‐infected mice, *Muc5ac* mRNA and bronchoalveolar lavage Fluid (BALF) MUC5AC levels were significantly reduced in the mouse lungs but MUC5B did not show the same changes as MUC5AC.

Gefitinib is an EGFR TKI that is often used clinically for chemotherapy in non‐small cell lung cancer (NSCLC). Studies have shown that it can reduce GCH in mice, suggesting that gefitinib may be a useful treatment option for mucus hypersecretion [29]. Guan et al. [30] also reached the same conclusion in an acrolein‐induced mouse model. In addition, they also found that gefitinib could inhibit the expression of MUC5AC and EGFR in the lungs. In a mouse model of asthma, research has found that EGFR activation reduced Claudin 1 expression while increasing MUC5AC expression in both in‐vitro and in‐vivo models [31]. Erlotinib attenuates allergic airway inflammation in mice by restoring Claudin 1 expression and reducing MUC5AC expression. These findings suggest that claudin 1 reduction caused using EGFR activation promotes GC metaplasia and that restoring claudin 1 using EGFR inhibition can prevent this. In addition, erlotinib can inhibits EGFR phosphorylation and downstream signaling pathways to reduce mucus production [32].

According to Currier et al. [33], EFGR inhibitors have also shown therapeutic value in viral infections. Their results demonstrate that an EGFR inhibitor (erlotinib) significantly decreased RSV A2 2‐20 F‐induced airway mucin expression in BALB/c mice. According to a single‐center retrospective study published in 2020, the first‐line EGFR TKIs gefitinib and afatinib were found to inhibit airway mucus hypersecretion earlier and more significantly than the inhibition of tumor growth in patients with lung malignancies treated as targeted agents [34].

An inhibitor of the EGFR downstream signaling pathway, Bio‐11006 has clinical potential for the treatment of mucus hypersecretion and is currently in Phase IIb trials [35]. Moreover, recent research suggests that targeting the EGFR signaling pathway is a promising therapeutic option for COVID‐19 [36].

Although EGFR inhibitors have shown therapeutic value, their side effects remain a concern. According to a safety and efficacy trial on the EGFR inhibitors (BIBW 2948) by Woodruff, the EGFR‐TKI had poor drug tolerance and did not significantly reduce mucin storage in airway epithelial cells of patients with COPD. BIBW 2948 subjects were more likely than those of the control group to discontinue treatment due to side effects (24.0% vs. 4.3%) [37]. Many EGFR‐TKIs have common adverse reactions such as skin disease, abdominal distension, paronychia, oral mucositis, liver injury, and interstitial lung disease. Studies have found that, compared to erlotinib and gefitinib, afatinib has greater toxicity and is associated with a higher incidence of adverse reactions. According to the parallel trial results of Thomas et al., gefitinib has a better safety profile than erlotinib in patients with NSCLC despite having similar effectiveness. This suggests that erlotinib is a superior option among EGFR inhibitors [38].

Currently, there are no successful EGFR‐TKI trials in human asthma and patients with COPD [39]. To consider EGFR‐TKI as a treatment option for patients with mucus hypersecretion, we need to develop EGFR‐TKI with improved efficacy and measure the potential adverse effects of EGFR signal inhibition on airway mucus hypersecretion on other systems. At the same time, small molecule inhibitors frequently suffer from issues such as secondary drug resistance [31], low solubility, a propensity to bind to plasma albumin, and unpredictable pharmacokinetics. More clinical research is required to assess the efficiency and safety of EGFR inhibition on airway mucus hypersecretion.

## 7 Summary

Airway mucus hypersecretion is a key pathophysiological hallmark of bronchopneumonic illnesses, including various types of lung inflammation, COPD, asthma, and pulmonary CF. It is a common manifestation and plays an important role in promoting the occurrence and development of the disease and is also a factor affecting the prognosis of patients.

Mucus hypersecretion is a complex communication network formed by the interaction of various cells, cytokines, and different factors and pathways, in which EGFR plays a central regulatory role. The conventional treatment for hypersecretion, including both pharmacological and nonpharmacological treatment, are not fully effective. Monoclonal antibodies and small molecule tyrosine kinases, which inhibit airway mucus hypersecretion by blocking airway GC metaplasia and mucus gland hyperplasia, are considered important targets in drugs for mucus hypersecretion. Current research suggests that erlotinib is a superior option among EGFR inhibitors because it has higher safety and less toxicity. Notably, blocking the activation of EGFR will inhibit airway inflammation, airway submucosal collagen deposition, and airway mucus secretion, thereby improving asthmatic lung function and providing a new weapon for the treatment of asthma, especially severe asthma.

A thorough comprehension of the EGFR pathway's function in controlling the secretion of mucus from the airways will yield novel insights into the diagnosis, prognosis, and management of a variety of respiratory diseases. Currently, some targeted therapies have demonstrated curative effects, but more research is still needed to determine an appropriate administration strategy, dosage, and safety. Targeted EGFR nanotherapy offers a promising approach for treating hypersecretory diseases but may have serious side effects due to nonspecificity. Therefore, developing drug molecules with low off‐target activity to maximize airway selectivity and combining them with appropriate drug delivery device design will be an important step in reducing unnecessary side effects and advancing the clinical application of EGFR inhibitors.

## Author Contributions

Yuanyuan Zhang drafted and revised the original manuscript. Yuanyuan Zhang, Fuping Yang, and Chunyu Yan completed the design and production of the figure together. Enmei Liu and Yu Deng are accountable for all aspects of the work and ensure that questions related to the accuracy or integrity of any part of the work are appropriately investigated and resolved. Each author fulfills related requirements and accepts the final submission.

## Ethics Statement

It was waived by the institution review board due to the nature of review.

## Conflicts of Interest

Prof. Enmei Liu is the Deputy Editor‐in‐Chief of *Pediatric Discovery*. To minimize bias, she was excluded from all editorial decision‐making related to the acceptance of this article for publication. The remaining authors declare no conflicts of interest.

## Data Availability

The data that support the findings of this study are available from the corresponding author upon reasonable request.
